# An overview of a 4-year period of admissions of young people with eating difficulties to a general admissions unit

**DOI:** 10.1192/bjb.2025.6

**Published:** 2025-06

**Authors:** Victoria Thomas, Amy Wright, Jessica Jobling, David O'Sullivan

**Affiliations:** 1 Associate specialist child and adolescent psychiatrist working in the Specialist Children and Young Peoples Inpatient Service, Ferndene Hospital, Prudhoe, UK; 2 Assistant psychologist working in the Specialist Children and Young Peoples Inpatient Service, Ferndene Hospital, Prudhoe, UK; 3 Honorary assistant psychologist working in the Specialist Children and Young Peoples Inpatient Service, Ferndene Hospital, Prudhoe, UK; 4 Consultant clinical psychologist and approved clinician, working in the Specialist Children and Young Peoples Inpatient Service, Ferndene Hospital, Prudhoe, UK

**Keywords:** Anorexia nervosa, child and adolescent psychiatry, in-patient treatment, feeding or eating disorders, service development

## Abstract

**Aims and method:**

To review and explore the eating disorder admissions to an in-patient child and adolescent mental health hospital which had restarted taking such presentations. This was done by conducting three audits using RiO (an electronic patient records system) and including all young people with eating disorders or related difficulties admitted between 1 February 2019 and 30 June 2023. As part of this, relevant practice standards were identified using the baseline assessment tool in UK national guidelines.

**Results:**

The audits identified 46 completed admissions, detailing demographic information, nasogastric and restraint feeding, therapeutic interventions and medication, admission and discharge routes, length of admission and more.

**Clinical implications:**

The review highlighted the apparent overall success of a general admission unit in treating eating disorders and related difficulties and identified key areas of importance and development in terms of clinical practice.

Eating disorders are complex psychiatric disorders characterised by negative beliefs about the self, eating, body shape and weight, which in turn can lead to behaviours such as restricted oral intake, binge eating and compensatory behaviours such as excessive exercise or vomiting. Common eating disorders include anorexia nervosa, bulimia nervosa, binge eating disorder and other specified feeding or eating disorders.^1^ Eating disorders significantly disrupt social, psychological and physical functioning.^2^ Social consequences include social isolation, poor quality of life and social withdrawal. Psychological consequences include negative cognitions that maintain eating difficulties, comorbid difficulties such as anxiety, and suicide. Physical complications include malnutrition, electrolyte imbalances, osteoporosis^1^ and even death; anorexia nervosa has the highest mortality rate of any mental health condition by both natural and unnatural causes.^3,4^ Eating disorders typically have onset in adolescence and disproportionately affect young people, specifically females;^4–6^ those aged 13–17 years are at highest risk for development of an eating disorder and only 25% of those with an eating disorder are male.^1^

Overall, an estimated 1.25 million people in the UK have a formal eating disorder diagnosis,^7^ although the number of young people presenting to eating disorder services is increasing.^8^ Such increase is suggested to have been further exacerbated by the COVID-19 pandemic, with the UK seeing double the number of adolescents referred to National Health Service (NHS) eating disorder services during this time.^9^ The COVID-19 pandemic has worsened various risk factors of eating disorders at a societal level through social isolation, food insecurity, uncertainty, loss of routine, disruption to accessing face-to-face services, pressures to exercise and challenges to lose weight.^10^

## Treatment

In the UK, national guidelines maintain that the majority of young people presenting with an eating disorder should be managed within competent out-patient services. In-patient treatment is indicated in a minority of cases owing to acute medical or psychiatric need or where out-patient treatment has been ineffective.^1^ When in-patient care is indicated, guidelines state the following priorities: that in-patient care should be individualised, accounting for the specific needs of the presenting adolescent; care should be provided by a multidisciplinary team (MDT) with expertise in eating disorders; care should be provided in an age-appropriate unit near the adolescent's home; and evidence-based psychological therapies should be delivered by clinicians with experience working with eating disorders, alongside nutritional rehabilitation and physical health monitoring.^1^

When indicated, in-patient treatment provided by specialist eating disorder units (SEDUs) is often reported to result in positive treatment outcomes, such as healthy weight gain at discharge and a decrease in symptoms of general psychopathology.^11^ However, admissions to SEDUs have also been associated with adverse psychosocial outcomes, including social learning of disordered eating behaviours among peers and feelings of disconnection from the individual's existing community support network.^12^ The duration of in-patient stay is typically long, especially for those requiring nasogastric feeding or detention using mental health legislation.^13^ To mitigate social disconnection, promote collaborative care and facilitate smooth transfer back into the community, this long period of admission further highlights the need for care to be provided in close proximity to a young person's community support network.^1^ However, access to local specialist in-patient care can be a challenge, with a high demand on beds that have been historically unevenly distributed and difficult to access in several areas of the UK.^9,14^ These factors likely contribute to individuals presenting with eating disorders also accounting for a significant proportion of admissions to non-specialised units such as generic psychiatric units and acute paediatric wards.^15,16^

## CNTW eating disorder services for children and adolescents

Cumbria, Northumberland, Tyne and Wear NHS Foundation Trust (CNTW) includes community eating disorder services for children and young people established in 2011. However, owing to the commissioning arrangements in place between 2011 and 2019, no in-patient service for this age group provided specialist eating disorder care within the trust's locality during this time period. As a result, young people were placed out of area (on average 88 miles away from their support network), creating significant challenges for collaborative care and transitioning back into the community.

Following a full options appraisal, enhanced eating disorders care was commissioned within the locally established CNTW children and young people's general admissions unit (GAU), complemented by local acute paediatric provision. The unit provides access to a full MDT, including psychology, speech and language therapy, occupational therapy, art therapy, dietetics and psychiatry, and access to on-site education and structured day activities. Patients also have access to an independent advocate. Patients admitted to the eating disorder pathway have a personalised meal plan (which can include nasogastric feeding when indicated) and receive meal support delivered predominantly by nursing assistants. There is a weekly MDT meeting, regular formal care coordination meetings, as well as frequent meetings to review care plans with the patient and family, dietician, responsible clinician and community teams. Patients are supported to access increasing home leave as progress allows. There is a service level agreement with the local acute trust to allow close working with paediatrics, and a comprehensive training programme for staff working on the eating disorders pathway.

## The current study

The aim of the project outlined in this paper was to retrospectively gather demographic data relating to admissions to the unit of young people with eating disorders/difficulties and to use the baseline assessment tool in the National Institute for Health and Care Excellence (NICE) clinical guideline on the recognition and treatment of eating disorders^1^ to identify standards that were relevant to practice in the in-patient unit.

## Method

Three audit registrations – one initial audit and two subsequent re-audits – were conducted using RiO (an electronic patient records system) to identify all patients with the primary presenting difficulty of an eating disorder or disordered eating that had completed an admission to Ferndene (an in-patient child and adolescent mental health hospital). The audit periods were 1 February 2019 to 15 April 2020, 15 April 2020 to 5 May 2022, and 5 May 2022 to 30 June 2023, spanning almost a 4.5-year period.

This research was registered with the CNTW audit team. Ethical approval and patient consent were not required for this research.

## Results

A total of 46 completed admissions were identified. These admissions were completed by 41 different young people, as 5 individuals completed 1 re-admission during the time period. Age at admission ranged from 12 to 17 years and all except two were female admissions. Most individuals were admitted directly from the family home (28) and then from acute paediatric wards (17), with 1 coming from a SEDU. Most were detained under the Mental Health Act 1983 during at least part of their admission (39). Most individuals had a previous admission to an acute hospital ward (39) and some had a previous admission to a mental health ward (10). Length of previous involvement with respective community services ranged from 2 weeks to 3 years 5 months. The average percentage weight for height (a measure of body weight as a percentage of ideal weight given height, gender and age) on admission was 82.3%.

During their admission, 28 individuals (28/46; 60.9%) required nasogastric feeding at some point, 19 of whom (19/46; 41.3%) required the use of approved restraint to administer the feed at some point. Almost all admissions had dietetics involvement (45), with the family being involved in dietetic reviews in 38 of these cases. Patients were offered a range of different therapeutic interventions, including psychology (34/46), family therapy (20/46), dialectical behaviour therapy (18/46) and art psychotherapy (15/46). Out of the 46 admissions, 33 received some form of medication (72%); the remaining 13 did not receive any medication (28%). Selective serotonin reuptake inhibitors (24/46) and olanzapine (20/46) were mostly used, with ‘as required’ medication used to manage distress in some cases (10/46). With regard to physical health monitoring, 43 admissions received regular blood monitoring throughout their admission and 32 received an electrocardiogram. No patients were referred for a bone scan. Table 1 gives admission information on gender, age, Mental Health Act status, nasogastric feeding and medication.

**Table 1:** Admission information

	Admissions (*n* = 46)	
	*n*	%
Gender		
Female	44	95.65
Male	2	4.35
Age at admission, years		
12	4	8.70
13	6	13.04
14	6	13.04
15	9	19.57
16	11	23.91
17	9	19.57
Detained under Mental Health Act		
Yes	39	84.78
No	7	15.22
Nasogastric feeding		
Yes	28	60.9
Under restraint	19	41.3
Medication		
Antidepressants	24	52.17
Olanzapine	20	43.48
As required medication	10	21.74
No medication	13	28.26

On discharge, the average percentage weight for height was 92.3% – an increase of 10% compared with the average admission weight for height. Of the 46 admissions, 27 (58.7%) had a primary diagnosis of anorexia nervosa on discharge, 8 (17.4%) had another eating disorder primary diagnosis, 9 (19.6%) had a non-eating disorder primary mental health diagnosis and 2 (4.3%) had a presentation that was felt to best fit with that of pervasive arousal withdrawal syndrome (PAWS), which is a child psychiatric condition characterised by pervasive refusal to eat or drink, as well as talk, walk and engage in any self-care, where symptoms cannot be accounted for by any other condition.^17^ In total, 43 of the admissions were discharged to their family home address, 2 were discharged to care homes and 1 was transferred to a specialist PAWS unit.

**Fig. 1:**
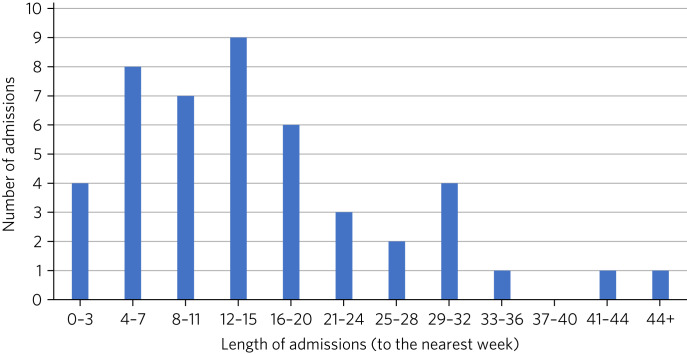
Length and number of admissions.

Overall average length of stay was around 16 weeks (Fig. 1). The length of stay for admissions that received nasogastric feeding (*n* *=* 28) ranged from 3 to 75 weeks, with an average length of stay of just over 20 weeks. When excluding the two patients with a diagnosis of PAWS, the length of stay for admissions who received nasogastric feeding ranged from 3 to 35 weeks, with an average length of stay of just over 17 weeks. The length of stay for admissions who did not require nasogastric feeding (*n* *=* 18) ranged from 0.5 to 25 weeks, with an average length of stay of just under 9 weeks.

## Discussion

The initial audit was completed at a time when the general adolescent in-patient service began to admit young people with eating disorders/disordered eating as their primary presentation, after a period of approximately 8 years of not doing so. Prior to 2011, the trust's GAU had admitted young people with this presentation and had significant expertise in managing these disorders. The changes in commissioning that resulted in in-patient beds moving to a SEDU in an adjacent NHS trust coincided with the formation of the local children and young people's specialist community eating disorder teams. This resulted in many of the in-patient staff who had expertise and interest in managing eating disorders moving to the community team. In 2019, when the commissioning arrangements changed again, the clinical team consisted of many staff across the MDT who did not have any significant experience of assessing or treating eating disorders. The in-patient service worked hard, in conjunction with the specialist community teams, to implement appropriate training and supervision arrangements to quickly build staff's confidence and expertise.

The nature of what the service was able to provide changed over the 4-year audit period, as ongoing training was developed and implemented. Initially, GAU nursing staff were unable to insert nasogastric tubes, which necessitated patients being transferred to the acute paediatric ward when nasogastric feeding was required. Comprehensive training in the siting and use of nasogastric tubes means that patients no longer need to be transferred to the acute hospital, unless there are physical health risks. The in-patient unit developed, and continues to benefit from, a close working relationship with the local paediatric hospital.

### Service and clinical implications

The data have been helpful in providing assurance to staff that, despite the perception that managing eating disorders/disordered eating is ‘new’ to them and very challenging, they are providing a service that is following relevant NICE guidelines and having a positive effect. The data are positive in terms of achieving significant weight restoration and having an average length of stay which is less than that on other in-patient units for adolescents.^13^ In other studies patients have reported that in-patient stays create feelings of isolation and disconnectedness.^18^ Having a unit placed within a trust's locality allows family collaboration and also helps in terms of home leave and transitioning back into the community.

The data support the notion that detention under the Mental Health Act and nasogastric feeding are associated with increased length of stay.^13^ It is also of note that the two longest admissions both presented with PAWS, something a GAU is less familiar with and less equipped to support fully.

It is worth noting that the NICE guidelines on eating disorders detail treatment recommendations for patients with anorexia nervosa, bulimia nervosa and binge eating disorder. Only 58.7% of our admissions had a confirmed diagnosis of anorexia nervosa; 23.9% did not have a diagnosis of any type of eating disorder. Where patients present with disordered eating as a symptom of a mental disorder (other than a primary or typical eating disorder), then clear formulation of the underlying difficulties is crucial in ensuring that they receive the most appropriate care, as well as managing any physical risk associated with the disordered eating. Positively, it is evident from the audit that patients were able to access a range of different therapeutic approaches during their admissions.

This review of admissions over a 4-year period has helped us to consolidate areas of key learning and further shape and develop our clinical practice. These are that: (a) with the right scaffolding and support structures in place a GAU can successfully support and treat young people with eating disorder presentations; (b) a specific MDT with specialist skills is needed and a means of training/up-skilling the staff team; (c) it is important to establish and maintain a positive working relationship with local specialist eating disorder teams and paediatric teams; (d) nasogastric feeding under restraint has a substantial impact on young people and staff and it requires a system in place to validate and support this; (e) there is a need to routinely review and monitor the potential iatrogenic harm of hospital admissions and to balance positive risk-taking with the actual risk of death/physical harm; and (f) additional support and research are needed regarding establishing the best method of care in relation to the increasing numbers of autism, PAWS and disordered eating presentations.

## Data Availability

The data that support the findings of this study are available from the corresponding author on reasonable request.
